# First report of gastric muscle-retracting sign identification and endoscopic intermuscular dissection

**DOI:** 10.1055/a-2801-5227

**Published:** 2026-03-19

**Authors:** Andrea Sorge, Anne Hoorens, Michele Montori, Maria Eva Argenziano, Lobke Desomer, Edoardo Vincenzo Savarino, David James Tate

**Affiliations:** 1 60200 Department of Gastroenterology and Hepatology, University Hospital Ghent, Ghent, Belgium; 2 9308 Department of Surgery, Oncology and Gastroenterology, University of Padua, Padua, Italy; 3 18624 Gastroenterology Unit, Azienda Ospedale Università di Padova, Padua, Italy; 4 60200 Department of Pathology, University Hospital Ghent, Ghent, Belgium; 5 9294 Clinic of Gastroenterology, Hepatology and Emergency Digestive Endoscopy, Università Politecnica delle Marche, Ancona, Italy; 6 26656 Faculty of Medicine and Health Sciences, Ghent University, Ghent, Belgium; 7 192827 Gastroenterology and Hepatology, AZ Delta vzw, Roeselare, Belgium

**Fig. 1:**
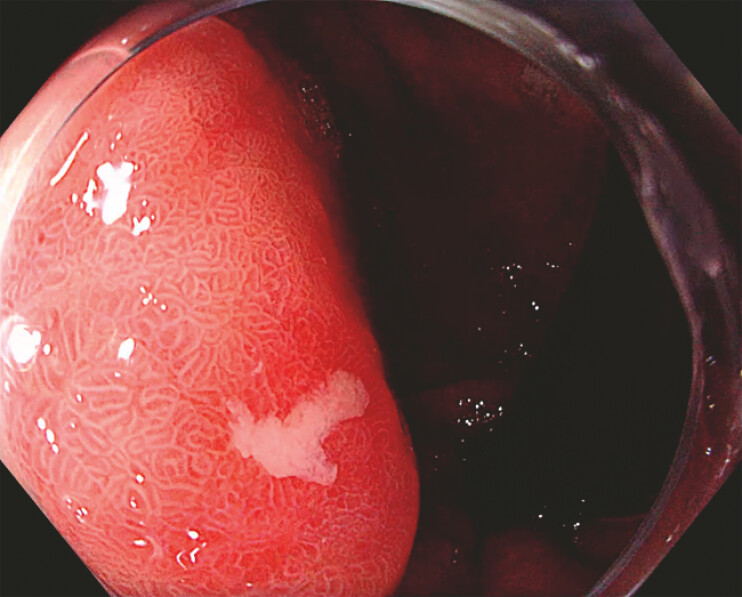
Slightly elevated gastric lesion, with a central erosion (Paris 0-IIa+c), measuring 8 mm located in the proximal stomach of a patient with chronic autoimmune gastritis, compatible with a neuroendocrine tumor.

**Fig. 2:**
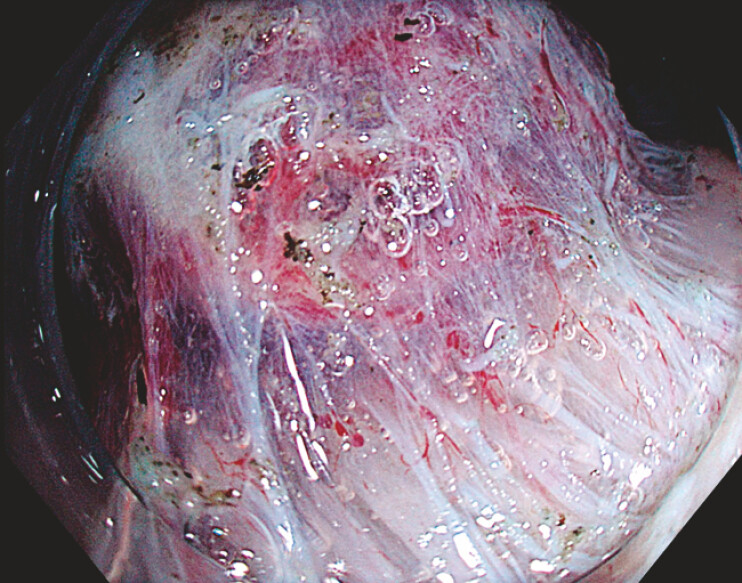
Muscle-retracting sign. Submucosal invasion and fibrosis causing tethering of the muscularis propria to the overlying lesion, narrowing of the submucosal space, and non-staining submucosa. Appearance under CO2 insufflation with maximal exposure of the dissection plane secondary to the application of traction.

**Fig. 3:**
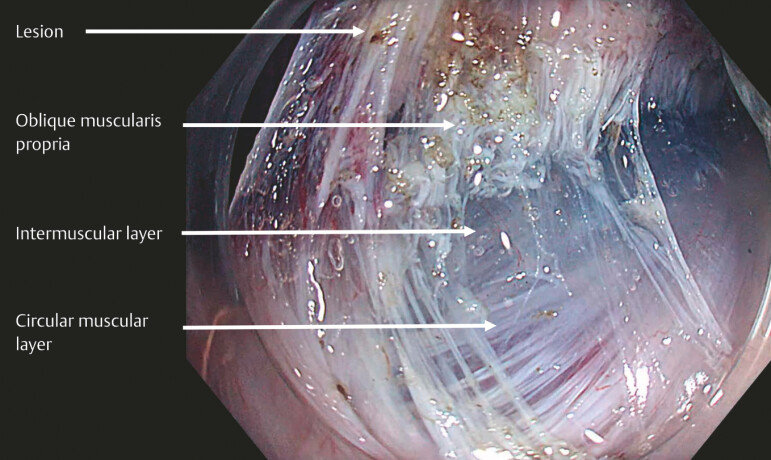
The appearance of the resection defect following the incision of the oblique layer of the muscularis propria. The intermuscular dissection plane between the oblique and the circular layers of the muscularis propria is exposed by applying multipoint elastic traction with the pulley technique.

**Fig. 4:**
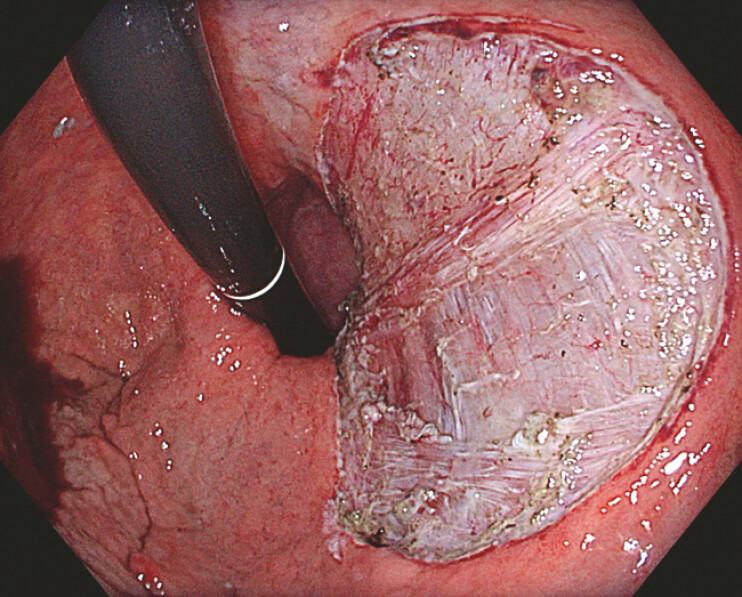
Gastric endoscopic intermuscular dissection defect showing the exposed circular and oblique muscle layers.

**Fig. 5:**
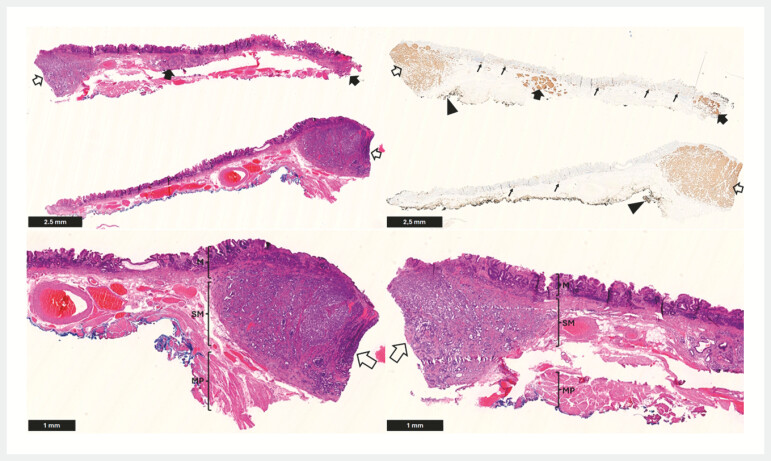
Top left: Cross-section of the resection specimen divided into two halves (H&E). A large well-differentiated neuroendocrine tumour (NET) extends deeply into the submucosa (unfilled arrow). Two additional smaller NETs are present (filled arrows), one of which reaches the lateral mucosal margin. Top right: Synaptophysin immunohistochemistry of the same cross-section. The large NET extends deeply into the submucosa (unfilled arrow). The deep resection margin is free of tumour (filled arrowhead). Two smaller NETs (filled arrows), including one involving the lateral mucosal margin, and extensive micronodular enterochromaffin-like (ECL) cell hyperplasia (thin arrows) are also identified. Bottom left and bottom right: Higher magnification views of the left and right halves of the section shown in the top left panel. In both, the large NET extends deeply into the submucosa (unfilled arrows) with the presence of the oblique muscularis propria layer free of neoplastic cells. The gastric corpus mucosa demonstrates chronic atrophic gastritis with intestinal metaplasia. In combination with the micronodular ECL-cell hyperplasia, this suggests autoimmune gastritis (M: mucosa; SM: submucosa; MP: muscularis propria).

A 70-year-old woman with severe chronic atrophic autoimmune gastritis was referred for evaluation of an 8-mm gastric lesion located in the proximal stomach ( Fig. 1 ). Staging endoscopic ultrasound suggested a T1 type 1 gastric neuroendocrine tumor (NET) without nodal metastasis. After a multidisciplinary team discussion, the patient agreed on endoscopic resection. During endoscopic submucosal dissection (ESD) with multipoint elastic traction ( Video 1 ), a muscle-retracting sign (MRS) was observed ( Fig. 3 ), with the lesion adherent to the oblique muscularis propria and loss of the dissection plane for ESD 1 . Therefore, a deeper resection plane was selected by accessing the intermuscular space between the oblique and the circular muscularis propria ( Fig. 2 ), which is more easily identifiable in the proximal stomach 2 . Gastric endoscopic intermuscular dissection (EID) was performed en bloc ( Fig. 4 ). Histopathology revealed an R0 resection of a well-differentiated 12 mm NET G2 invading deeply into the submucosa (pT2 according to the American Joint Committee on Cancer Staging System, version 9 3 ) without lymphovascular invasion in the context of atrophic gastritis ( Fig. 5 ). Two smaller G1 NETs, including one involving the lateral margin, were identified in the same specimen. Given the radical resection and the patientʼs age and preference, the multidisciplinary team agreed on surveillance with endoscopy and imaging.

Gastric endoscopic intermuscular dissection achieving a radical (R0) resection of an early T2 neuroendocrine tumor.Video 1

At the surveillance endoscopy performed 5 months after the EID, no recurrence was seen at the EID scar, and three G1 NETs <5 mm were endoscopically resected.

To our knowledge, this is the first description of gastric MRS and one of the earliest reports of gastric EID. Gastric MRS may serve as a warning sign during endoscopic resection, suggesting the presence of deep submucosal invasion where ESD is unlikely to be radical. In highly selected cases, EID may be considered in the stomach to achieve R0 resection when the oblique layer of the muscularis propria is visible 4 .

Endoscopy_UCTN_Code_TTT_1AO_2AG_3AD
